# SARS-CoV-2 Infection in School Settings, Okinawa Prefecture, Japan, 2021–2022

**DOI:** 10.3201/eid3011.240638

**Published:** 2024-11

**Authors:** Yoshihiro Takayama, Yusuke Shimakawa, Ryota Matsuyama, Gerardo Chowell, Ryosuke Omori, Tetsuharu Nagamoto, Taro Yamamoto, Kenji Mizumoto

**Affiliations:** Okinawa Chubu Hospital, Uruma, Japan (Y. Takayama); Okinawa Prefecture Epidemiological Statistics and Analysis Committee, Naha-shi, Japan (Y. Takayama, Y. Shimakawa, R. Matusuyama, R. Omori, T. Nagamoto, K. Mizumoto); Institute of Tropical Medicine Department of International Health and Medical Anthropology, Nagasaki University, Nagasaki, Japan (Y. Takayama, T. Yamamoto); Institut Pasteur, Université Paris Cité, Paris, France (Y. Shimakawa); Pasteur International Unit at Kumamoto University/National Center for Global Health and Medicine, Tokyo, Japan (Y. Shimakawa); Rakuno Gakuen Univerisity, Ebetsu, Japan (R. Matsuyama); Georgia State University School of Public Health, Atlanta, Georgia, USA (G. Chowell); International Institute for Zoonosis Control, Hokkaido University, Sapporo, Japan (R. Omori); Kyoto University Graduate School of Advanced Integrated Studies in Human Survivability, Kyoto, Japan (K. Mizumoto)

**Keywords:** COVID-19, SARS-CoV-2, viruses, respiratory infections, zoonoses, vaccine-preventable diseases, school health, secondary infection, school closure, educational loss, Okinawa Prefecture, Japan

## Abstract

During the COVID-19 pandemic, widespread school closures were implemented globally based on the assumption that transmission among children in the school environment is common. However, evidence regarding secondary infection rates by school type and level of contact is lacking. Our study estimated the frequency of SARS-CoV-2 infection in school settings by examining the positivity rate according to school type and level of contact by using data from a large-scale school-based PCR project conducted in Okinawa, Japan, during 2021–2022. Our results indicate that, despite detection of numerous positive cases, the average number of secondary infections remained relatively low at ≈0.5 cases across all types of schools. Considering the profound effects of prolonged closures on educational access, balancing public health benefits against potential long-term effects on children is crucial.

During the COVID-19 pandemic, many countries implemented extensive school closures from nurseries to high schools to reduce infection transmission within schools and curb community spread. School closures have proven effective against highly transmissible diseases (*1*–*3*). Closures may target specific classrooms, grades, or entire schools when positive cases are confirmed, and, in some instances, regional closures are enacted to contain outbreaks or curb epidemic spread.

During school closures, the shift to online learning places new demands on both students and teachers, particularly those unfamiliar with digital teaching methods. In addition, the cost of acquiring necessary online learning devices falls on families, posing a financial barrier for some. The prolonged closures during the COVID-19 pandemic have resulted in substantial learning setbacks; students lost ≈1.5 school years by the end of 2021. This lapse has led to a concerning rise in learning poverty; 70% of 10-year-olds cannot read and comprehend simple stories, especially in East Asia, South Asia, the Middle East, and Latin America (*4*). Face-to-face learning not only maximizes educational opportunities but also plays a crucial role in preventing the exacerbation of poverty issues. Moreover, the closure of nurseries and elementary schools has substantial socioeconomic effects, frequently necessitating parental leave to care for children (*5*).

The rationale for school closures rests on the assumption of high child-to-child transmission within schools. Both schools and households, where persons gather in close proximity for extended periods, are identified as high-risk settings for transmission (*6*). Some studies have suggested that reducing classroom sizes or increasing the distance between student seats can effectively reduce transmission rates (*7*). However, large-scale research on the extent of COVID-19 transmission among students, who now exhibit increased hygiene awareness and mask usage compared with prepandemic times, remains limited. Moreover, detailed data on the actual number of secondary infections that occur when positive cases occur in schools are scarce.

Okinawa Prefecture in Japan implemented the prefecture-wide School PCR Project to mitigate infection spread. Under this initiative, if a child or student tested positive for SARS-CoV-2, all persons within the affected class were subject to reverse transcription PCR (RT-PCR) testing. Those tested were then required to remain home until they received negative results. Although previous studies have reported positivity rates among contacts (*8*,*9*), data on the number of secondary infections per school and the extent of testing, stratified by school types and contact levels, are lacking.

In this study, we sought to estimate the frequency of SARS-CoV-2 infection in school settings by examining positivity rates according to school type and contact level. The goal was to guide the optimal implementation of school closures, balancing public health concerns with the children’s educational needs.

## Methods

### Study Setting

Okinawa Prefecture, situated at the southwestern tip of the Japan archipelago and isolated by sea, comprises 1.17% of Japan’s population of 14.47 million. In Okinawa, the School PCR Project ran from May 2021 to September 2022. We initially targeted all the elementary schools (n = 268), junior high schools (n = 149), high schools (n = 67), special support schools (n = 22), and after-school children’s clubs (n = 501). In September 2021, the scope was expanded to include all nurseries (n = 456) and kindergartens (n = 303) in Okinawa (*10*–*12*). We excluded vocational training schools (n = 3) because of their small number and classification challenges. Since March 24, 2022, no close contacts have been identified, and after June 2022, the project’s focus was narrowed down to support schools, after-school children’s clubs, and nurseries and kindergartens (*13*,*14*).

The after-school children’s clubs serve elementary school students whose parents work late and whose grandparents live far away. Those clubs typically use vacant classrooms or children’s halls in elementary schools. During school closures of elementary schools affected by COVID-19, the clubs accommodated students during daytime hours (*15*,*16*).

**Figure 1:**
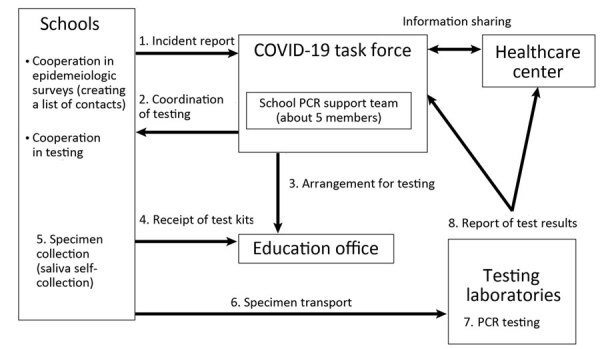
Overview of the SARS-CoV-2 PCR project conducted in school settings, Okinawa Prefecture, Japan, 2021–2022. The school’s PCR Project protocol begins when the Prefectural COVID-19 Task Force is notified of a SARS-CoV-2 infection in a student. The prefectural school PCR support team will arrange for testing, with students providing saliva samples through self-collection of saliva. School officials receive test containers from the education office and transport the specimens to the testing laboratories. Contacts are generally all children or students in the class with a positive case, and close contacts are those within 1 meter from the index case-patient for >15 minutes without appropriate infection control measures.

The School’s PCR Project protocol (Figure 1) begins when the Prefectural COVID-19 Task Force is notified of a SARS-CoV-2 infection in a student. The prefectural school PCR support team will arrange for testing, and students provide saliva samples through self-collection of saliva. School officials receive test containers from the education office and transport the specimens to the testing laboratories. Contacts are generally all children or students in the class with a positive case, and close contacts are those who were within 1 meter of the index case-patient for >15 minutes without the appropriate infection control measures (*17*). Initially, contacts and close contacts were placed on home leave until test results were known (in the case of contacts) and for 7 days from the last exposure (in the case of close contacts). After March 24, 2022, no attendance suspension was required in elementary, junior high, and high schools for contacts. Close contacts remained in school if they were asymptomatic (*18*). Parental consent was obtained for the PCR tests (*17*); saliva testing was not invasive, and refusals from testing because of concerns about infection were rare. Samples were centrally tested at the following local laboratories using PCR: Anti Viral Screening System, Okinawa Institute of Science and Technology, SRL, Okinawa PCR Testing Center, Okinawa Prefecture Environmental Science Center, Okinawa Institute of Environmental Conservation, and Okinawa Clinical Laboratory Center.

During the study period, the predominant variants in Okinawa Prefecture shifted over time. The Alpha variant was dominant during May–June 2021, followed by the Delta variant during July 2021–November 2021, and then the Omicron variant from December 2021 onward (*19*).

### Data Sources

The School PCR Project collected data on each event, including facility name, name of the index case-patient, date of birth, date of last contact between index case-patient and contacts, date of illness onset of index case-patient, lists of students who had contact with the index case-patient during the infectious period, and contact location (*16*). In this analysis, provided data included the date of illness onset of the index case-patient, type of school, health center with jurisdiction over that school, number of identified contacts with an index case-patient, and number of tested positives among contacts with information on the level of contacts from the Okinawa Prefecture government. We divided contacts into 2 groups by contact level: those in close contact (close contacts) and those in contact except for close contacts (non–close contacts) with the index case-patient.

### Ethics Considerations

The School PCR project complied with Japan law and Okinawa Prefecture government policy. As part of the public health response to COVID-19, this study involved the secondary analysis of anonymized data provided by Okinawa Prefecture. The study was exempt from institutional review board review because no information in the dataset could directly or indirectly identify persons.

### Statistical Analysis

This analysis defined the positivity rate as the proportion of contacts testing positive among those exposed to index case-patients. A negative binomial regression model with a log link identified factors associated with higher positivity rates among all contacts (close and non-close contacts) and close contacts. By setting the number of positive cases as a response variable and the number of tested students as an offset, we used the following school PCR project-related variables as explanatory variables: the type of school, health center with jurisdiction over that school, RT-PCR testing institute, and whether the PCR tests performed in the target school were the first implementation or subsequent implementations. We conducted multivariate analyses by using the explanatory variables, which showed a p value <0.20, as determined by the Wald test in the univariate analysis. We examined multicollinearity by using the variance inflation factor. We excluded variables exceeding the variance inflation factor value of 4.0 one by one from the multivariate analyses. We stratified data by whether they involved the school’s first or subsequent RT-PCR test, reflecting increased infection control awareness after the initial outbreak. We performed all statistical analyses in R version 4.2.3 (The R Project for Statistical Computing, https://www.r-project.org).

## Results

**Figure 2:**
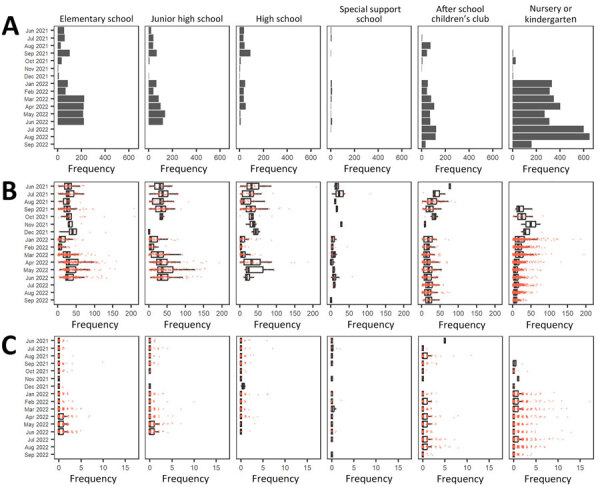
Results of SARS-CoV-2 PCR projects conducted in school settings, by month and school type, Okinawa Prefecture, Japan, 2021–2022. A) Number of events that results in school contact tracing, by month and school type. B) Number of PCR tests per event, by month stratified by school type. C) Number of secondary SARS-CoV-2 infection cases per event, by month and school type. Box-and-whisker plots indicate the median (solid black lines), the first and third quartiles (box left and right edges), and minimum and maximum values excluding outliers (error bars). Orange particles represent each observed datapoint.

We calculated the number of events by school type and month (Figure 2, panel A; Appendix Table 1). The total number of events for the entire period was 6,736; of those, 1,311 (19.5%) occurred at elementary schools, 721 (10.7%) at junior high schools, 392 (5.8%) at high schools, 67 (1.0%) at special support schools, 830 (12.3%) at after-school children’s clubs, and 3,415 (50.7%) at nurseries and kindergarten. Overall, the number of events increased dramatically after January 2022. Relative to the 2021 average, the increases in January 2022 were 108% for elementary schools, 161% for junior high schools, 54% for high schools, 271% for special support schools, 172% for after-school children’s clubs, and 4,483% for nurseries and kindergarten.

We calculated the number of PCR tests per event by school type and month during June 2021–September 2022 (Figure 2, panel B; Appendix Table 1). In total, we identified 143,184 contacts, of whom 20,546 were classified as close contacts and all of whom underwent PCR tests. The median number of PCR tests per event was 15.0 (interquartile range [IQR] 6.0–29.0) for all tested schools for the entire period. By school type, the median number of PCR tests per event was 27.0 (IQR 19.0–40.0) for elementary schools, 31.0 (IQR 16.0–47.0) for junior high schools, 20.0 (IQR 4.0–37.0) for high schools, 8.0 (IQR 3.0–14.5) for special support schools, 18.0 (IQR 8.0–28.0) for after-school children’s clubs, and 9.0 (IQR 4.0–18.0) for nurseries and kindergartens. With a few exceptions, the median number of PCR tests per event declined dramatically after January 2022. The relative reduction in medians from 2021 to 2022 was 76% in elementary schools, 83% in junior high schools, 86% in senior high schools, 78% in special support schools, 36% in after-school children’s clubs, and 52% in nurseries and kindergartens.

We calculated the number of secondary infections per event by school type and month for June 2021–September 2022 (Figure 2, panel C; Appendix Table 1). Overall, the mean number of secondary infections per event was 0.43 (median 0, IQR 0–0) for all tested schools during the entire period. By school type, the mean number of secondary infections per event was 0.34 (median 0, IQR 0–0) for elementary schools, 0.35 (median 0, IQR 0–0) for junior high schools, 0.28 (median 0, IQR 0–0) for high schools, 0.13 (median 0, IQR 0–0) for special support schools, 0.50 (median 0, IQR 0–1) for after-school children’s clubs, and 0.49 (median 0, IQR 0–0) for nurseries and kindergartens. During January–February 2022, the mean number of secondary infections was much higher than the overall average for the period in 2021, having a relative increase of 47% in January 2022 and 104% in February 2022.

After we excluded the months when the number of events was small (<10 events/month/school type), the top 2 months with the highest average number of secondary infections per event were June 2022 (0.50) and April 2022 (0.45) for elementary schools, February 2022 (0.50) and May 2022 (0.58) for junior high schools, January 2022 (0.53) and April 2022 (0.40) for high schools, February 2022 (0.17) and June 2022 (0.17) for special support schools, February 2022 (0.68) and March 2022 (0.71) for nurseries and kindergartens, and July 2022 (0.67) and August 2022 (0.75) for after-school children’s clubs. By school type, the widest monthly range of secondary infections for elementary school was 0–10 in September 2021; for junior high schools, 0–10 in February 2022; for high schools, 0–7 in July 2021; for special support schools, 0–2 in July 2021 and February 2022; for after-school children’s clubs, 0–11 in August 2021; and for nurseries and kindergartens, 0–17 in February 2022.

**Figure 3:**
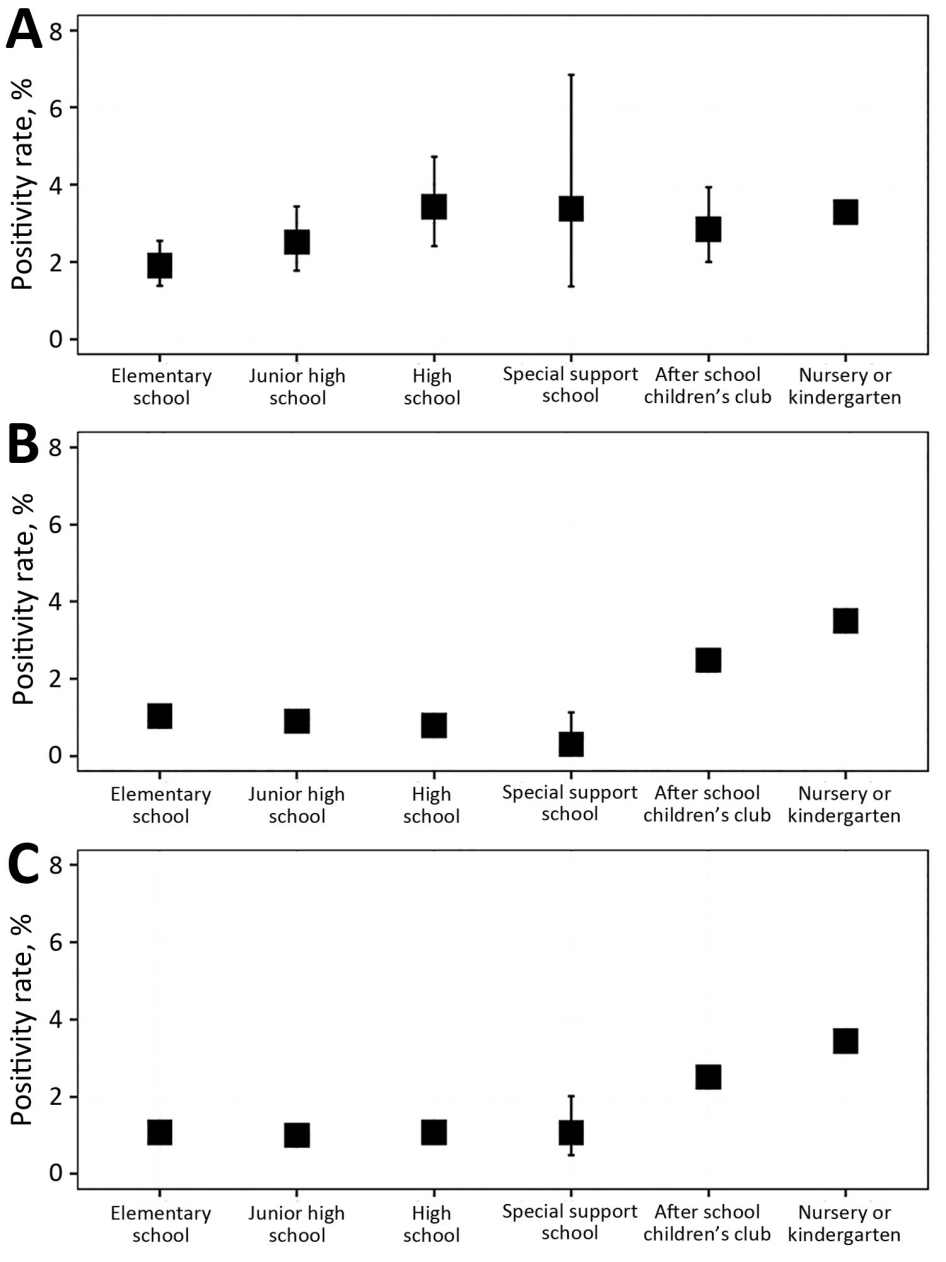
SARS-CoV-2 positivity rates in school settings among close contacts (A), non–close contacts (B), and all contacts (C) of infected children, by school type, Okinawa Prefecture, Japan, 2021–2022. In all panels, boxes indicate mean positivity rate and error bars indicate 95% CIs.

We stratified positive rates among contacts by school type by the level of contacts (Figure 3; Appendix Table 2). Positivity rates among all contacts were 2.04% (95% CI 1.96%–2.11%) (2,914/143,184), among close contacts were 3.06% (95% CI 2.83%–3.31%) (629/20,546), and among non–close contacts were 1.86% (95% CI 1.79%–1.94%) (2,285/122,638). Among all contacts, the top 2 highest positivity rates were 3.44% (95% CI 3.28%–3.60%) (1,676/48,472) in nurseries and kindergartens and 2.51% (95% CI 2.27%–2.76%) (416/16,592) in after-school children’s clubs, whereas this rate was ≈1.0% in other school types. Mean positivity rates among close contacts in all school types ranged from 2.0 to 3.5, with no significant differences. Among non–close contacts, the top 2 highest positivity rates were 3.50% (95% CI 3.31%–3.70%) (1,205/34,421) in nurseries and kindergartens and 2.48% (95% CI 2.24%–2.74%) (381/15,362) in after-school children’s clubs, and the lowest positivity rate was 0.31% (95% CI 0.04–1.13) (2/637) in special support schools.

Regarding the proportion of the positives in close contacts among the positives in all contacts (Appendix Table 3), nurseries and kindergartens and high schools had the highest values: 74.6% (95% CI 71.0%–78.03%) for nurseries and kindergartens and 41.7% (95% CI 31.0%–52.9%) for high schools, excluding special support schools because of data scarcity. This proportion maintained relatively high values after January 2022.

We calculated the result from the regression analysis of positivity rates among all contacts on the basis of various factors (Appendix Tables 4, 5). Compared with elementary school, positivity rates were significantly lower in high school (relative risk [RR] 0.61, 95% CI 0.46–0.81; p<0.001) and special support school (RR 0.59, 95% CI 0.43–0.80; p<0.01) and significantly higher in after-school children’s club (RR 1.34, 95% CI 1.05–1.72; p = 0.02), after adjusting for the first event, school type, area, and period. Compared with PCR tests in October 2021, PCR tests conducted starting in January 2022 were associated with significant increases in positivity rates: a 155% (95% CI 39%–382%) increase in January 2022, a 339% (95% CI 140%–728% [significant]) increase in February 2022, a 242% (95% CI 89%–537%) increase in March 2022, a 231% (95% CI 83%–516%) increase in April 2022, a 215% (95% CI 73%–491%) increase in May 2022, and a 237% (95% CI 86%–532%) increase in June 2022.

We calculated the results from the regression analysis of positivity rates among close contacts as a function of the variables (Appendix Tables 6, 7). Compared with October 2021, the positivity rates of PCR tests were significantly higher for January 2022 (RR 2.66, 95% CI 1.06–7.08; p = 0.04), February 2022 (RR 5.42, 95% CI 2.17–14.38), March 2022 (RR 4.41, 95% CI 1.77–11.66), and April 2022 (RR 3.40, 95% CI 0.89–13.49) after adjusting for the first event, school type, area, and period.

## Discussion

This study assessed the effectiveness of screening tests in school PCR projects conducted in Okinawa Prefecture, Japan, during May 2021–September 2022. After January 2022, coinciding with the prevalence of the more infectious Omicron variant (*15*), the average number of secondary infections was almost the same or increased in all school types yet remained well below 1. Our findings suggest that, in schools where appropriate infection-control measures were in place, the risk for secondary infection was not necessarily high, and the actual status of infection in school settings should be considered when implementing school closures. In addition to households, schools have been cited as a major route of infection for children, and measures such as class closures and school closures have been deployed when even a limited number of positive cases were confirmed. In Japan, after the spread of the novel coronavirus, handwashing and hand sanitizing have been routinely encouraged, and wearing masks for elementary school students and older essentially became mandatory. Furthermore, during the study period, mokushoku (“silent eating”) was recommended by the government, and students were prohibited from talking during lunch meals (*20*,*21*). This analysis shows that schools with appropriate infection-control measures are not always at high risk. Field epidemiologic investigations corroborate this low infection risk at schools, indicating that the transmission route constitutes only 4% of elementary schools, 6% of junior high schools, and 4% of high schools (*22*). In addition, students identified as close contacts with the infected person are required to stay at home for 7 days from the date of the last contact with the infected person (*23*). Given those findings, alternatives to canceling classes might include allowing only close contacts to stay home or requiring testing for all contacts, requiring only those testing positive to quarantine. Such approaches could also be considered for managing other infectious diseases, such as influenza.

During the study period, secondary infection risks were significantly higher at nurseries and kindergartens and after-school children’s clubs. In nurseries and kindergartens, factors such as inappropriate masking and physical proximity because of younger ages probably contributed to increased risk. Despite overlapping age groups with elementary schools, after-school clubs showed significantly higher infection risks, suggesting factors unique to these settings (e.g., smaller spaces per child and higher population density when nonregular attendees join) exacerbate transmission risks. Moreover, during school closures, many younger elementary students, particularly from households with essential workers or dual-income parents unable to take leave, are sent to these clubs (*15*,*16*). Many after-school clubs in Okinawa Prefecture are privately operated and involve low usage of public facilities such as school spaces (*24*). Activities common in clubs, such as communal meal preparation, may also increase contact intensity and infection risk. Although the clubs are crucial for supporting working parents and are integral to social infrastructure, the challenges of enforcing strict infection controls in such environments suggest that flexible, rather than restrictive, measures are preferable during outbreaks.

Given that secondary infection risks vary by school type and level of contact, differences should be carefully considered when narrowing down the target population, especially under budget constraints. Depending on the type of school, the importance of RT-PCR testing for non–close contacts may need to be enhanced. Although nurseries and kindergartens and after-school children’s clubs consistently showed high risks for all contacts and non–close contacts, we observed no significant differences among close contacts across school types. Of note, in after-school children’s clubs, the positivity rate for non–close contacts occasionally exceeded that for close contacts. Furthermore, the absolute number of test-positive cases among non–close contacts was higher than among close contacts.

The multivariate analysis results, which showed that both the close contact and all contacts positivity rates were significantly higher after January 2022 compared with October 2021, are consistent with the higher infectivity of the Delta variant. We reconfirmed that the positivity rate for all contacts was significantly higher in after-school children’s clubs than in elementary schools. The significantly lower positive rates in high and special-needs schools may be because of the students and their guardians’ higher awareness of infection-control measures.

The relatively high percentage of positive test results among close contacts over all positive results after January 2022 is because of the effect of numerous reported cases of infection attributable to the outbreak of the contagious Omicron strain (Figure 2, panel A). This development led to a restriction on the number of tests performed on non–close contacts.

One limitation of our study is that the average number of secondary infections is well below 1.0. However, false negatives might have occurred, considering that the test was performed only once and the RT-PCR test has relatively low sensitivity using saliva specimens (*25*). Nevertheless, although an alternative, nasal swab tests also present disadvantages; of note, false-negative results may be more common in children because of the difficulty in obtaining adequate specimens (*26*). Second, because of the prolonged COVID-19 epidemic, unofficial reports indicated that some junior and high school students did not stay home but instead chose to socialize with friends during school closure periods. In addition, cases of infection might have occurred not in the classroom but in extracurricular activities involving physical contact. However, our analysis does not reflect these cases because of the lack of data. In addition, secondary infections include cases contracted outside of school, which is influenced by changes in local community prevalence. The secondary infection rate we report represents the maximum value, encompassing infections acquired outside school. The estimated value would probably be even lower if external cases were excluded. Furthermore, Japan’s commuting and class systems differ from those in other countries, potentially affecting the generalizability of these findings. In Japan, students typically walk or ride bicycles to school instead of using school buses, and teachers, rather than students, move between classrooms because of the home-class system (*27*). Moreover, students eat lunch in their home classroom with their teachers, rather than in a cafeteria, as is common in the United States (*28*).

In summary, our analysis indicates that, during the 2021–2022 period of the COVID-19 pandemic, many positive cases were reported in school settings among students, with varying risks of infection by school type and the level of contact. However, the average number of secondary infections was generally low, limited to ≈0.5 cases across all school types. Remarkably, no large-scale child-to-child transmission occurred, a prerequisite for school closure. Because prolonged school closure deprives students of educational opportunities and leads to education poverty issues, it is worth reconsidering the target population and the measures to be taken. In addition, national and local governments could consider enhancing control measures in after-school children’s clubs to improve safety.

Appendix Additional information about SARS-CoV-2 infection in school settings, Okinawa Prefecture, Japan, 2021–2022.
