# Supplementary material for: SARS-CoV-2 Infection in School Settings, Okinawa Prefecture, Japan, 2021–2022
Source: Emerg Infect Dis. 2024 Nov;30(11):2343–51. doi: 10.3201/eid3011.240638 (PMC11521161; doi:10.3201/eid3011.240638)
Supplement: Appendix — Additional information about SARS-CoV-2 infection in school settings, Okinawa Prefecture, Japan, 2021–2022. [file 24-0638-Techapp-s1.pdf]

Article DOI: https://doi.org/10.3201/eid3011.240638

EID cannot ensure accessibility for supplementary materials supplied by authors. Readers who have difficulty accessing supplementary content should contact the authors for assistance.

SARS-CoV-2 Infection in School Settings, Okinawa Prefecture, Japan, 2021–2022

Appendix

Appendix Table 1. No. PCR tests per event and secondary cases identified per event by school type and month, May 31, 2021–September 30, 2022, Okinawa, Japan.

	No. PCR tests														
	School type														
	Elementary school		Junior high school		High school		Special support school		After-school children’s club		Nursery, Kindergarten§		All schools	
Time	No. events	Median, IQR	No. events	Median, IQR	No. events	Median, IQR	No. events	Median, IQR	No. events	Median, IQR	No. events	Median, IQR	No. events	Median, IQR
Entire period	1311	27.0, 19.0-40.0	721	31.0, 16.0-47.0	392	20.0, 4.0-37.0	67	8.0, 3.0-14.5	830	18.0, 8.0-28.0	3415	9.0, 4.0-18.0	6736	15.0, 6.0-29.0
Jun 2021	53	29.0, 19.0-37.0	21	31.0, 16.0-39.0	36	32.5, 20.0-52.0	5	15.0, 12.0-21.0	1	77.0, 77.0-77.0	NA	NA	116	30.0, 17.0-40.0
Jul 2021	59	29.0, 23.0-44.0	39	38.0, 28.0-52.5	32	28.5, 6.8-37.8	8	23.0, 14.5-34.5	5	35.0, 32.0-49.0	NA	NA	143	30.0, 22.5-45.0
Aug 2021	26	26.5, 16.0-31.0	38	31.5, 10.3-39,5	40	10.0, 3.8-30.3	1	12.0, 12.0-12.0	75	27.0, 17.0-40.0	0	NA	180	24.5, 12.0-36.3
Sep 2021	101	26.0, 17.0-33.0	68	35.0, 19.5-46.3	90	30.5, 18.0-42.0	2	17.0, 15.5-18.5	44	21.0, 12.0-31.0	4	17.5, 12.3-29.3	309	28.0, 16.0-41.0
Oct 2021	33	29.0, 24.0-37.0	7	33.0, 30.5-37.5	9	33.0, 26.0-37.0	0	NA	7	35.0, 28.5-38.5	26	25.0, 14.3-35.8	82	30.0, 24.0-36.8
Nov 2021	4	30.5, 30.0-39.5	0	NA	4	36.5, 28.8-41.5	1	29.0, 29.0-29.0	2	9.0, 8.5-9.5	2	49.5, 36.3-62.8	13	30.0, 23.0-40.0
Dec 2021	10	40.5, 31.8-51.0	1	1.0, 1.0-1.0	2	44.0, 38.0-50.0	0	NA	0	NA	4	34.0, 30.5-46.3	17	35.0, 31.0-52.0
Jan 2022	85	7.0, 4.0-20.0	65	6.0, 3.0-24.0	47	4.0, 2.0-13.5	9	4.0, 2.0-10.0	52	18.0, 6.0-29.0	330	16.0, 7.0-32.0	588	12.5, 5.0-27.0
Feb 2022	66	4.0, 2.0-10.5	40	5.0, 2.0-13.0	33	3.0, 2.0-4.0	12	4.0, 3.0-6.0	44	16.0, 7.0-20.3	313	15.0, 7.0-24.0	508	11.0, 4.0-19.0
Mar 2022	222	25, 15.0-33.0	85	25.0, 7.0-40.0	35	6.0, 3.0-14.5	7	8.0, 2.0-12.0	79	13.0, 6.0-21.5	347	18.0, 8.0-28.5	775	19.0, 7.0-30.0
Apr 2022	219	30, 23.0-55.0	101	32.0, 22.0-50.0	50	32.5, 12.0-55.8	3	1.0, 1.0-6.0	106	16.0, 7.0-23.0	401	9.0, 4.0-17.0	880	19.0, 7.0-37.0
May 2022	213	28.0, 23.0-49.0	137	37.0, 25.0-66.0	6	24.0, 18.0-63.8	3	8, 6.5-10.0	70	19.0, 5.3-28.0	270	6.0, 3.0-13.8	699	21.0, 7.0-37.0
Jun 2022	220	26, 21.8-42.3	119	34.0, 24.0-52.5	8	18.5, 16.5-27.3	12	7.5, 5.8-13.8	72	13.0, 7.0-26.3	310	7.0, 3.0-15.0	741	19.0, 7.0-31.0
Jul 2022	NA	NA	NA	NA	NA	NA	3	9.0, 7.5-11.5	122	18.0, 11.0-29.0	600	9.0, 4.0-16.0	725	10.0, 5.0-18.0
Aug 2022	NA	NA	NA	NA	NA	NA	0	NA	118	17.5, 8.0-25.0	648	7.0, 3.0-12.0	766	8.0, 4.0-15.0
Sep 2022	NA	NA	NA	NA	NA	NA	1	1.0, 1.0-1.0	33	19.0, 11.0-27.0	160	6.0, 3.0-11.0	194	7.0, 4.0-14.0

	No. secondary infections													
	School type													
	Elementary school		Junior high school		High school		Special support school		After-school children’s club		Nursery, Kindergarten§		All schools	
Time	Mean	Median, IQR	Mean	Median, IQR	Mean	Median, IQR	Mean	Median, IQR	Mean	Median, IQR	Mean	Median, IQR	Mean	Median, IQR
Entire period	0.34	0 (0-0)	0.35	0 (0-0)	0.28	0 (0-0)	0.13	0 (0-0)	0.50	0 (0-1)	0.49	0 (0-0)	0.43	0 (0-0)
Jun 2021	0.15	0 (0-0)	0.24	0 (0-0)	0.39	0 (0-0)	0.00	0 (0-0)	5.00	5 (5-5)	NA	NA	0.28	0 (0-0)
Jul 2021	0.10	0 (0-0)	0.44	0 (0-0)	0.34	0 (0-0)	0.38	0 (0-0.25)	0.20	0 (0-0)	NA	NA	0.27	0 (0-0)
Aug 2021	0.15	0 (0-0)	0.13	0 (0-0)	0.28	0 (0-0)	0.00	0 (0-0)	0.71	0 (0-1)	NA	NA	0.41	0 (0-0)
Sep 2021	0.27	0 (0-0)	0.09	0 (0-0)	0.10	0 (0-0)	0.00	0 (0-0)	0.39	0 (0-0)	0.50	0 (0-0.5)	0.20	0 (0-0)
Oct 2021	0.33	0 (0-0)	0.00	0 (0-0)	0.22	0 (0-0)	NA	NA	0.14	0 (0-0)	0.15	0 (0-0)	0.22	0 (0-0)
Nov 2021	0.00	0 (0-0)	NA	NA	0.00	0 (0-0)	0.00	0 (0-0)	0.00	0 (0-0)	1.00	1 (1-1)	0.15	0 (0-0)
Dec 2021	0.20	0 (0-0)	0.00	0 (0-0)	0.50	0.50 (0.25-0.75)	NA	NA	NA	NA	0.00	0 (0-0)	0.18	0 (0-0)

	No. PCR tests													
	School type													
	Elementary school		Junior high school		High school		Special support school		After-school children’s club		Nursery, Kindergarten§		All schools	
Time	No. events	Median, IQR	No. events	Median, IQR	No. events	Median, IQR	No. events	Median, IQR	No. events	Median, IQR	No. events	Median, IQR	No. events	Median, IQR
Jan 2022	0.22	0 (0-0)	0.22	0 (0-0)	0.53	0 (0-0)	0.00	0 (0-0)	0.27	0 (0-0)	0.47	0 (0-1)	0.39	0 (0-0)
Feb 2022	0.12	0 (0-0)	0.50	0 (0-0)	0.15	0 (0-0)	0.17	0 (0-0)	0.43	0 (0-1)	0.68	0 (0-1)	0.54	0 (0-0)
Mar 2022	0.34	0 (0-0)	0.33	0 (0-0)	0.17		0.29	0 (0-0.5)	0.25	0 (0-0)	0.71	0 (0-1)	0.49	0 (0-0)
Apr 2022	0.45	0 (0-1)	0.32	0 (0-0)	0.40	0 (0-0)	0.00	0 (0-0)	0.56	0 (0-1)	0.49	0 (0-0)	0.46	0 (0-0)
May 2022	0.36	0 (0-1)	0.58	0 (0-1)	0.50	0 (0-0)	0.00	0 (0-0)	0.43	0 (0-1)	0.33	0 (0-0)	0.40	0 (0-1)
Jun 2022	0.50	0 (0-1)	0.39	0 (0-1)	0.25	0 (0-0)	0.17	0 (0-0)	0.28	0 (0-0)	0.38	0 (0-0)	0.40	0 (0-0)
Jul 2022	NA	NA	NA	NA	NA	NA	0.00	0 (0-0)	0.67	0 (0-1)	0.57	0 (0-1)	0.59	0 (0-1)
Aug 2022	NA	NA	NA	NA	NA	NA	NA	NA	0.75	0 (0-1)	0.42	0 (0-0)	0.47	0 (0-1)
Sep 2022	NA	NA	NA	NA	NA	NA	0.00	0 (0-0)	0.21	0 (0-0)	0.23	0 (0-0)	0.22	0 (0-0)

§RT-PCR screening for nursery school/kindergarten started September 18, 2021, while screening for other schools began April 26, 2021. Since March 24, 2022, close contacts had not been identified, and after June 2022, this project was conducted only in special support schools, after-school children’s clubs and nurseries/kindergartens.

Appendix Table 2. Result of screening tests in School PCR Projects by school type stratified by level of contact, 2021-2022, Okinawa, Japan

		Close contacts			Non-close contacts			All contacts		
School type	No. schools/clubs	N	Pos	%Pos (95% CI)	N	Pos	%Pos (95% CI)	N	Pos	%Pos (95% CI)
Total	6736	20546	629	3.06 (2.83-3.31)	122638	2285	1.86 (1.79-1.94)	143184	2914	2.04 (1.96-2.11)
Elementary School	1311	2263	43	1.90 (1.38-2.55)	39406	409	1.04 (0.94-1.14)	41669	452	1.08 (0.99-1.19)
Junior high school	721	1476	37	2.51 (1.77-3.44)	23730	215	0.91 (0.79-1.03)	25206	252	1.00 (0.88-1.13)
High school	392	1049	36	3.43 (2.41-4.72)	9082	73	0.80 (0.63-1.01)	10131	109	1.08 (0.88-1.30)
Special support school	67	207	7	3.38 (1.37-6.84)	637	2	0.31 (0.04-1.13)	844	9	1.07 (0.49-2.01)
After-school children’s clubs	830	1230	35	2.85 (1.99-3.94)	15362	381	2.48 (2.24-2.74)	16592	416	2.51 (2.27-2.76)
Nursery school, Kindergarten[1)]	3415	14321	471	3.29 (3.00-3.59)	34421	1205	3.50 (3.31-3.70)	48472	1676	3.44 (3.28-3.60)

§RT-PCR screening for nursery school/kindergarten started September 18, 2021, while screening for other schools began April 26, 2021. Since March 24, 2022, close contacts had not been identified, and after June 2022, this project was conducted only in special support schools, after-school children’s clubs and nurseries/kindergartens.
N: Number

Appendix Table 3. A proportion of the positives in close contacts among the positives in all contacts by school type and by month in screening tests in School PCR Projects, 2021-2022, Okinawa, Japan.

	School type					
	Elementary school	Junior high school	High school	Special support school	After-school children's clubs	Nursery school, Kindergarten§
Time	Percent (95% CI)	Percent (95% CI)	Percent (95% CI)	Percent (95% CI)	Percent (95% CI)	Percent (95% CI)
Total	25.8 (19.3-33.1)	39.0 (29.1-49.5)	41.7 (31.0-52.9)	100.0 (59.0-100.0)	26.9 (19.5-35.4)	74.6 (71.0-78.03)
Jun, 2021	12.5 (0.3-52.7)	20.0 (0.5-71.6)	14.3 (1.8-42.8)	NA	100.0 (47.8-100.0)	NA
Jul, 2021	33.3 (4.3-77.7)	23.5 (6.8-49.9)	0.0 (0.0-28.5)	100.0 (29.2-100.0)	100.0 (2.5-100.0)	NA
Aug, 2021	0.0 (0.0-60.2)	0.0 (0.0-52.2)	36.4 (10.9-69.2)	NA	13.2 (5.5-25.3)	NA
Sep, 2021	18.5 (6.3-38.1)	0.0 (0.0-45.9)	22.2 (2.8-60.0)	NA	35.3 (14.2-61.7)	100.0 (15.8-100.0)
Oct, 2021	9.1 (0.2-41.3)	NA	0.0 (0.0-84.2)	NA	100.0 (2.5-100.0)	100.0 (39.8-100.0)
Nov, 2021	NA	NA	NA	NA	NA	50.0 (1.3-98.7)
Dec, 2021	50.0 (1.3-98.7)	NA	0.0 (0.0-97.5)	NA	NA	NA
Jan, 2022	47.4 (24.5-71.1)	21.4 (4.7-50.8)	76.0 (54.9-90.6)	NA	35.7 (12.8-64.9)	52.9 (44.7-61.0)
Feb, 2022	92.9 (66.1-99.8)	95.0 (75.1-99.9)	100.0 (47.8-100.0)	100.0 (15.8-100.0)	26.3 (9.2-51.2)	86.4 (81.0-90.7)
Mar, 2022	14.5 (7.5-24.4)	35.7 (18.6-55.9)	50.0 (11.8-88.2)	100.0 (15.8-100.0)	25.0 (8.7-49.1)	77.7 (72.0-82.8)

§RT-PCR screening for nursery school/kindergarten started September 18, 2021, while screening for other schools began April 26, 2021. Since March 24, 2022, close contacts had not been identified.

Appendix Table 4. Factors associated with a positive rate (secondary cases/ all contacts) per event, October 2021-June 2022, Okinawa Prefecture, Japan

	Event			Univariate analysis		Multivariate analysis	
Variables	No	%	Mean positive rate (95% CI)	RR (95% CI)	P-value	RR (95% CI)	P-value
Event frequency							
First event	1238	28.8	0.024 (0.022-0.026)	1.0 (Reference)	-	1.0 (Reference)	NA
Second or more event	3055	71.2	0.017 (0.016-0.018)	0.73 (0.62-0.85)	<0.001***	0.96 (0.81-1.15)	0.66
School type							
Elementary school	431	10.0	0.021 (0.018-0.025)	1.0 (Reference)	-	1.0 (Reference)	NA
Junior high school	194	4.5	0.016 (0.012-0.020)	0.80 (0.51-1.24)	0.30	0.98 (0.63-1.53)	0.94
High school	1072	25.0	0.012 (0.011-0.013)	0.53 (0.41-0.69)	<0.001***	0.61 (0.46-0.81)	<0.001***
Special support school	553	12.9	0.011 (0.010-0.013)	0.54 (0.40-0.73)	<0.001***	0.59 (0.43-0.80)	<0.01**
Nursery/Kindergarten	47	1.1	0.014 (0.005-0.030)	0.64 (0.22-1.67)	0.38	0.69 (0.23-1.77)	0.46
After-school children’s club	1996	46.5	0.030 (0.028-0.032)	1.33 (1.04-1.70)	0.02*	1.34 (1.05-1.72)	0.02*
Area							
North	9	0.2	0.038 (0.005-0.130)	1.0 (Reference)	-	1.0 (Reference)	NA
Central	911	21.2	0.028 (0.026-0.031)	0.98 (0.14-8.74)	0.99	0.76 (0.12-6.54)	0.80
South	1409	32.8	0.019 (0.018-0.021)	0.66 (0.09-5.82)	0.70	0.56 (0.09-4.76)	0.57
Naha city	1640	38.2	0.014 (0.013-0.015)	0.50 (0.07-4.40)	0.52	0.43 (0.07-3.70)	0.42
Miyako	324	7.5	0.025 (0.021-0.029)	0.80 (0.11-7.12)	0.83	0.58 (0.09-5.02)	0.60
Ishigaki	NA						
Period							
Oct, 2021	82	1.9	0.006 (0.004-0.010)	1.0 (Reference)	-	1.0 (Reference)	NA
Nov, 2021	13	0.3	0.005 (0.001-0.016)	0.74 (0.10-4.05)	0.75	0.62 (0.08-3.17)	0.60
Dec, 2021	17	0.4	0.004 (0.001-0.012)	0.78 (0.15-3.42)	0.74	0.66 (0.13-2.75)	0.57
Jan, 2022	588	13.7	0.019 (0.017-0.022)	3.40 (1.82-6.52)	<0.001***	2.55 (1.39-4.82)	<0.01*
Feb, 2022	508	11.8	0.036 (0.032-0.040)	6.07 (3.26-11.64)	<0.001***	4.39 (2.40-8.28)	<0.001***
Mar, 2022	771	18.0	0.022 (0.020-0.024)	3.94 (2.14-7.47)	<0.001***	3.42 (1.89-6.37)	<0.001***
Apr, 2022	877	20.4	0.018 (0.016-0.020)	3.57 (1.94-6.76)	<0.001***	3.31 (1.83-6.16)	<0.001***
May, 2022	696	16.2	0.015 (0.013-0.016)	2.90 (1.56-5.53)	<0.01**	3.15 (1.73-5.91)	<0.001***
Jun, 2022	741	17.3	0.017 (0.015-0.019)	3.19 (1.72-6.06)	<0.001***	3.37 (1.86-6.32)	<0.001***

Asterisks denote the significance level according to the following P-values: ***P<0.001, **P<0.01, *P<0.05, ¶P<0.10
No: Number, RR: Relative risk

Appendix Table 5. Positivity rates among all contacts by school type and month, 2021-2022, Okinawa, Japan.

	Positivity rates among all contacts													
	School type													
	Elementary school		Junior high school		High school		Special support school		After-school children’s club		Nursery, Kindergarten§		All schools	
Time	Pos/N	%Pos (95% CI)	Pos/N	%Pos (95% CI)	Pos/N	%Pos (95% CI)	Pos/N	%Pos (95% CI)	Pos/N	%Pos (95% CI)	Pos/N	%Pos (95% CI)	Pos/N	%Pos (95% CI)
Entire period	452/41669	1.08 (0.99-1.19)	252/25206	1.00 (0.88-1.13)	109/10131	1.08 (0.88-1.30)	9/844	1.07 (0.49-2.01)	416/16592	2.51 (2.27-2.76)	1676/48472	3.44 (3.28-33.60)	2914/143184	2.04 (1.96-2.11)
Jun 2021	8/1603	0.50 (0.22-0.98)	5/596	0.84 (0.27-1.95)	14/1451	0.96 (0.53-1.61)	0/80	0.00 (0.00-4.51)	5/77	6.49 (2.14-14.51)	2/96	2.08 (0.25-7.32)	32/3807	0.84 (0.58-1.18)
Jul 2021	6/1909	0.31 (0.12-0.68)	17/1570	1.08 (0.63-1.73)	11/867	1.27 (0.64-2.26)	3/253	1.19 (0.25-3.43)	1/186	0.54 (0.01-2.96)	4/695	0.58 (0.16-1.47)	38/4785	0.79 (0.56-1.09)
Aug 2021	4/631	0.63 (0.17-1.62)	5/1191	0.42 (0.14-0.98)	11/714	1.54 (0.77-2.74)	0/12	0.00 (0.00-26.46)	53/2265	2.34 (1.76-3.05)	2/99	2.02 (0.25-7.11)	73/4813	1.52 (1.19-1.90)
Sep 2021	27/3028	0.89 (0.59-1.29)	6/2318	0.26 (0.10-0.56)	9/3000	0.30 (0.14-0.57)	0/34	0.00 (0.00-10.28)	17/939	1.81 (1.06-2.88)	0/171	0.00 (0.00-2.13)	61/9415	0.65 (0.50-0.83)
Oct 2021	11/1394	0.79 (0.39-1.41)	0/241	0.00 (0.00-1.52)	2/332	0.60 (0.07-2.16)	0/0	NA	1/223	0.45 (0.01-2.47)	155/8087	1.92 (1.63-2.24)	18/2885	0.62 (0.37-0.98)
Nov 2021	0/156	0.00 (0.00-2.34)	0/0	NA	0/135	0.00 (0.00-2.70)	0/29	0.00 (0.00-11.94)	0/18	0.00 (0.00-18.53)	213/5844	3.64 (3.18-4.16)	2/437	0.46 (0.06-1.64)
Dec 2021	2/494	0.40 (0.05-1.45)	0/1	0.00 (0.00-97.50)	1/88	1.14 (0.03-6.17)	0/0	NA	0/0	NA	247/7456	3.31 (2.92-3.74)	3/754	0.40 (0.08-1.16)
Jan 2022	19/1146	1.66 (1.00-2.58)	14/987	1.42 (0.78-2.37)	25/558	4.48 (2.92-6.54)	0/59	0.00 (0.00-6.06)	14/998	1.40 (0.77-2.34)	196/5435	3.61 (3.13-4.14)	227/11835	1.92 (1.68-2.18)
Feb 2022	14/515	2.72 (1.49-4.52)	20/354	5.65 (3.48-8.59)	5/140	3.57 (1.17-8.14)	2/95	2.11 (0.26-7.40)	19/687	2.77 (1.67-4.29)	90/2833	3.18 (2.56-3.89)	273/7635	3.58 (3.17-4.02)

Time	Positivity rates among all contacts – School type: Elementary school Pos/N		Junior high school Pos/N		High school Pos/N		Special support school Pos/N		After-school children's club Pos/N		Nursery, Kindergarten§ Pos/N		All schools Pos/N	
	Pos/N	%Pos (95% CI)	Pos/N	%Pos (95% CI)	Pos/N	%Pos (95% CI)	Pos/N	%Pos (95% CI)	Pos/N	%Pos (95% CI)	Pos/N	%Pos (95% CI)	Pos/N	%Pos (95% CI)
Mar 2022	76/5871	1.29 (1.02-1.62)	28/2298	1.22 (0.81-1.76)	6/403	1.49 (0.55-3.21)	2/54	3.70 (0.45-12.75)	20/1260	1.59 (0.97-2.44)	118/3382	3.49 (2.90-4.16)	379/17342	2.19 (1.97-2.41)
Apr 2022	99/9312	1.06 (0.86-1.29)	32/4162	0.77 (0.53-1.08)	20/2020	0.99 (0.61-1.53)	0/13	0.00 (0.00-24.71)	59/1893	3.12 (2.38-4.00)	344/7241	4.75 (4.27-5.27)	406/22835	1.78 (1.61-1.96)
May 2022	77/8182	0.94 (0.74-1.17)	79/6501	1.22 (0.96-1.51)	3/248	1.21 (0.25-3.49)	0/25	0.00 (0.00-13.72)	30/1314	2.28 (1.55-3.24)	269/6053	4.44 (3.94-4.99)	279/19103	1.46 (1.30-1.64)
Jun 2022	109/7428	1.47 (1.21-1.77)	46/4987	0.92 (0.68-1.23)	2/175	1.14 (0.14-4.07)	2/160	1.25 (0.15-4.44)	20/1259	1.59 (0.97-2.44)	36/1350	2.67 (1.87-3.67)	297/17391	1.71 (1.52-1.91)
Jul 2022	NA	NA	NA	NA	NA	NA	0/29	0.00 (0.00-11.94)	82/2502	3.28 (2.61-4.05)	2/96	2.08 (0.25-7.32)	426/9772	4.36 (3.96-4.78)
Aug 2022	NA	NA	NA	NA	NA	NA	0/0	NA	88/2282	3.86 (3.10-4.73)	4/695	0.58 (0.16-1.47)	357/8335	4.28 (3.86-4.74)
Sep 2022	NA	NA	NA	NA	NA	NA	0/1	0.00 (0.00-97.50)	7/689	1.02 (0.41-2.08)	2/99	2.02 (0.25-7.11)	43/2040	2.11 (1.53-2.83)

[§]RT-PCR screening for nursery school/kindergarten started September 18, 2021, while screening for other schools began April 26, 2021. Since March 24, 2022, close contacts had not been identified, and after June 2022, this project was conducted only in special support schools, after-school children's clubs and nurseries/kindergartens.
Pos: Positive, N: Number

Appendix Table 6. Factors associated with a positivity rate (secondary cases/ close contacts) per event, October 2021-June2022, Okinawa Prefecture, Japan

Variables	Events No	Events %	Mean positive rate (95% CI)	Univariate analysis RR (95% CI)	Univariate analysis P-value	Multivariate analysis RR (95% CI)	Multivariate analysis P-value
The Nth time event							
First	710	47.8	0.033 (0.030-0.037)	1.0 (Reference)	-	1.0 (Reference)	NA
Second or more	776	52.2	0.032 (0.028-0.036)	0.99 (0.75-1.29)	0.91	0.93 (0.72-1.20)	0.57
School type							
Elementary school	70	4.7	0.030 (0.017-0.047)	1.0 (Reference)	-	1.0 (Reference)	NA
Junior high school	107	7.2	0.048 (0.032-0.069)	1.21 (0.54-2.70)	0.71	1.49 (0.67-3.27)	0.32
High school	217	14.6	0.022 (0.016-0.031)	0.69 (0.34-1.36)	0.52	0.73 (0.37-1.44)	0.35
Special support school	146	9.8	0.033 (0.023-0.047)	0.92 (0.45-1.89)	0.87	0.77 (0.38-1.59)	0.48
Nursery/Kindergarten	26	1.7	0.032 (0.009-0.080)	0.92 (0.21-3.50)	0.72	0.92 (0.21-3.41)	0.91
After-school children's club	920	61.9	0.033 (0.030-0.036)	1.12 (0.62-2.01)	0.38	1.09 (0.62-1.93)	0.76
Area							
North	8	0.5	0.040 (0.001-0.204)	1.0 (Reference)	NA	1.0 (Reference)	NA
Central	462	31.1	0.040 (0.034-0.046)	1.08 (0.11-24.24)	0.26	1.13 (0.12-25.33)	0.93
South	361	24.3	0.044 (0.038-0.051)	1.15 (0.12-25.85)	0.32	1.17 (0.12-26.27)	0.91
Naha city	537	36.1	0.024 (0.021-0.027)	0.71 (0.07-15.93)	<0.01**	0.67 (0.07-15.15)	0.77
Miyako	118	7.9	0.024 (0.017-0.034)	0.71 (0.07-16.22)	0.93	0.73 (0.07-16.73)	0.82
Ishigaki	NA						0
Period							
Oct, 2021	35	2.4	0.015 (0.005-0.031)	1.0 (Reference)	0.14	1.0 (Reference)	NA
Nov, 2021	6	0.4	0.010 (0.000-0.053)	1.29 (0.13-12.33)	0.29	1.15 (0.12-10.25)	0.90
Dec, 2021	9	0.6	0.008 (0.008-0.041)	0.76 (0.08-5.51)	0.32	0.71 (0.08-4.97)	0.72
Jan, 2022	458	30.8	0.022 (0.019-0.027)	2.78 (1.10-7.40)	<0.01**	2.66 (1.06-7.08)	0.04*
Feb, 2022	459	30.9	0.042 (0.037-0.048)	5.33 (2.12-14.12)	0.01	5.42 (2.17-14.38)	<0.001***
Mar, 2022	499	33.6	0.034 (0.030-0.039)	4.27 (1.71-11.29)	0.21	4.41 (1.77-11.66)	<0.01**
Apr, 2022	20	1.3	0.032 (0.013-0.064)	3.03 (0.79-12.18)	0.69	3.40 (0.89-13.49)	0.08[¶]

Asterisks denote the significance level according to the following P-values: ***P<0.001, **P<0.01, *P<0.05, [¶]P<0.10
No: Number, RR: Relative risk
Data for May and June 2022 are unavailable because close contacts were no longer identified after May 2022 due to a change in the system.

Appendix Table 7. Positivity rates among close contacts by school type and month, 2021-2022, Okinawa, Japan.

	Positivity rates among close contacts														
	School type														
	Elementary school		Junior high school		High school		Special support school		After-school children’s club		Nursery, Kindergarten§		All schools		
Time	Pos/N	%Pos (95% CI)	Pos/N	%Pos (95% CI)	Pos/N	%Pos (95% CI)	Pos/N	%Pos (95% CI)	Pos/N	%Pos (95% CI)	Pos/N	%Pos (95% CI)	Pos/N	%Pos (95% CI)
Entire period	43/2263	1.90 (1.38-2.55)	37/1476	2.51 (1.77-3.44)	36/1049	3.43 (2.41-4.72)	7/207	3.38 (1.37-6.84)	35/1230	3.38 (1.37-6.84)	471/14321	3.29 (3.00-3.59)	629/20546	3.06 (2.83-3.31)
Jun 2021	1/172	0.58 (0.01-3.20)	1/50	2.00 (0.05-10.65)	2/141	1.42 (0.17-5.03)	0/16	0.00 (0.00-20.59)	5/77	6.49 (2.14-14.51)	NA	NA	9/456	1.97 (0.91-3.71)
Jul 2021	2/163	1.23 (0.15-4.36)	4/143	2.80 (0.77-7.01)	0/75	0.00 (0.00-4.80)	3/60	5.00 (1.04-13.92)	1/44	2.27 (0.06-12.02)	NA	NA	10/485	2.06 (0.99-3.76)
Aug 2021	0/44	0.00 (0.00-8.04)	0/161	0.00 (0.00-2.27)	4/121	3.31 (0.91-8.25)	0/3	0.00 (0.00-70.76)	7/407	1.72 (0.69-3.51)	NA	NA	11/736	1.49 (0.75-2.66)
Sep 2021	5/314	1.59 (0.52-3.68)	0/157	0.00 (0.00-2.32)	2/131	1.53 (0.19-5.41)	0/3	0.00 (0.00-70.76)	6/159	3.77 (1.40-8.03)	2/37	5.41 (0.66-18.19)	15/801	1.87 (1.05-3.07)
Oct 2021	1/42	2.38 (0.06-12.57)	0/1	0.00 (0.00-97.50)	0/24	0.00 (0.00-14.25)	0/0	NA	1/33	3.03 (0.08-15.76)	4/314	1.27 (0.35-3.23)	6/414	1.45 (0.53-3.13)
Nov 2021	0/0	NA	0/0	NA	0/18	0.00 (0.00-18.53)	0/14	0.00 (0.00-23.16)	0/8	0.00 (0.00-36.94)	1/62	1.61 (0.04-8.66)	1/102	0.98 (0.02-5.34)
Dec 2021	1/59	1.69 (0.04-9.09)	0/0	NA	0/8	0.00 (0.00-36.49)	0/0	NA	5/176	2.84 (0.93-6.50)	0/66	0.00 (0.00-5.44)	1/133	0.75 (0.02-4.12)
Jan 2022	9/400	2.25 (1.03-4.23)	3/217	1.38 (0.29-3.99)	19/293	6.48 (3.95-9.94)	0/31	0.00 (0.00-11.22)	5/177	2.82 (0.92-6.47)	82/4151	1.98 (1.57-2.45)	118/5268	2.24 (1.86-2.68)
Feb 2022	13/487	2.67 (1.43-4.52)	19/328	5.79 (3.52-8.90)	5/140	3.57 (1.17-8.14)	2/47	4.26 (0.52-14.54)	5/137	3.65 (1.20-8.31)	184/4198	4.38 (3.78-5.05)	228/5377	4.24 (3.72-4.81)
Mar 2022	11/582	1.89 (0.95-3.36)	10/410	2.44 (1.18-4.44)	3/92	3.26 (0.68-9.23)	2/33	6.06 (0.74-20.23)	0/12	0.00 (0.00-26.46)	192/5298	3.62 (3.14-4.16)	223/6552	3.40 (2.98-3.87)
Apr 2022¶	NA	NA	0/9	0.00 (0.00-33.63)	1/6	16.67 (0.42-64.12)	0/0	NA	NA	NA	6/195	3.08 (1.14-6.58)	7/222	3.15 (1.28-6.39)

§RT-PCR screening for nursery school/kindergarten started September 18, 2021, while screening for other schools began April 26, 2021. Since March 24, 2022, close contacts had not been identified, and after June 2022, this project was conducted only in special support schools, after-school children’s clubs and nurseries/kindergartens.

¶Reports outside the period are also tabulated for reference.

Pos: Positive, N: Number

Appendix Table 8. Positivity rates among non-close contacts by school type and month, 2021-2022, Okinawa, Japan.

	Positivity rates among non-close contacts														
	School type														
	Elementary school		Junior high school		High school		Special support school		After-school children’s club		Nursery, Kindergarten§		All schools		
Time	Pos/N	%Pos (95% CI)	Pos/N	%Pos (95% CI)	Pos/N	%Pos (95% CI)	Pos/N	%Pos (95% CI)	Pos/N	%Pos (95% CI)	Pos/N	%Pos (95% CI)	Pos/N	%Pos (95% CI)
Entire period	124/14484	0.86 (0.71-1.02)	58/8089	0.72 (0.54-0.93)	49/6645	0.74 (0.55-0.97)	0/409	0.00 (0.00-0.90)	95/5435	1.75 (1.42-2.13)	158/8322	1.90 (1.62-2.22)	484/433384	1.12 (1.02-1.22)
Jun 2021	7/1431	0.49 (0.20-1.01)	4/546	0.73 (0.20-1.87)	12/1310	0.92 (0.47-1.59)	0/64	0.00 (0.00-5.60)	0/0	0.00 (0.00-0.00)	0/59	0.00 (0.00-6.06)	23/3351	0.69 (0.44-1.03)
Jul 2021	4/1746	0.23 (0.06-0.59)	13/1427	0.91 (0.49-1.55)	11/792	1.39 (0.70-2.47)	0/193	0.00 (0.00-1.89)	0/142	0.00 (0.00-2.56)	0/381	0.00 (0.00-0.96)	28/4300	0.65 (0.43-0.94)
Aug 2021	4/587	0.68 (0.19-1.74)	5/1030	0.49 (0.16-1.13)	7/593	1.18 (0.48-2.42)	0/9	0.00 (0.00-33.63)	46/1858	2.48 (1.82-3.29)	1/37	2.70 (0.07-14.16)	62/4077	1.52 (1.17-1.95)
Sep 2021	22/2714	0.81 (0.51-1.22)	6/2161	0.28 (0.10-0.60)	7/2869	0.24 (0.10-0.50)	0/31	0.00 (0.00-11.22)	11/780	1.41 (0.71-2.51)	0/105	0.00 (0.00-3.45)	46/8614	0.53 (0.39-0.71)
Oct 2021	10/1352	0.74 (0.36-1.36)	0/240	0.00 (0.00-1.53)	2/308	0.65 (0.08-2.33)	0/0	NA	0/190	0.00 (0.00-1.92)	73/3936	1.85 (1.46-2.33)	12/2471	0.49 (0.25-0.85)
Nov 2021	0/156	0.00 (0.00-2.34)	0/0	0.00 (0.00-97.50)	0/117	0.00 (0.00-3.10)	0/15	0.00 (0.00-21.80)	0/10	0.00 (0.00-30.85)	29/1646	1.76 (1.18-2.52)	1/335	0.30 (0.01-1.65)
Dec 2021	1/435	0.23 (0.01-1.27)	0/1	NA	1/80	1.25 (0.03-6.77)	0/0	NA	0/0	NA	55/2158	2.55 (1.93-3.30)	2/621	0.32 (0.04-1.16)
Jan 2022	10/746	1.34 (0.64-2.45)	11/770	1.43 (0.72-2.54)	6/265	2.26 (0.84-4.86)	0/28	0.00 (0.00-12.34)	9/822	1.09 (0.50-2.07)	190/5240	3.63 (3.14-4.17)	109/6567	1.66 (1.36-2.00)
Feb 2022	1/28	3.57 (0.09-18.35)	1/26	3.85 (0.10-19.64)	0/0	0.00 (0.00-0.00)	0/48	0.00 (0.00-7.40)	14/510	2.75 (1.51-4.56)	90/2833	3.18 (2.56-3.89)	45/2258	1.99 (1.46-2.66)
Mar 2022	65/5289	1.23 (0.95-1.56)	18/1888	0.95 (0.57-1.50)	3/311	0.96 (0.20-2.79)	0/21	0.00 (0.00-16.11)	15/1123	1.34 (0.75-2.19)	118/3382	3.49 (2.90-4.16)	156/10790	1.45 (1.23-1.69)

§RT-PCR screening for nursery school/kindergarten started September 18, 2021, while screening for other schools began April 26, 2021. Since March 24, 2022, close contacts had not been identified, and after June 2022, this project was conducted only in special support schools, after-school children’s clubs and nurseries/kindergartens.

Pos: Positive, N: Number
